# Seminal Vesicle Abnormalities and Exploratory miR-664-5p and FOXO Findings in Aged Mice Following Long-Term Butyl Benzyl Phthalate Exposure

**DOI:** 10.3390/antiox15081021

**Published:** 2026-08-17

**Authors:** Seonhwa Hwang, Hyun Bon Kang, Dae Hyun Kim, Hyung Hoi Kim, Min Hi Park

**Affiliations:** 1 College of Pharmacy, Kyungsung University, Busan 48434, Republic of Korea; reseon17@naver.com (S.H.);; 2 Brain Busan 21 Plus Research Project Group, Kyungsung University, Busan 48434, Republic of Korea; 3 Department of Food Science & Technology, College of Natural Resources and Life Science, Pusan National University, Miryang 50463, Republic of Korea; 4 Department of Medicine, School of Medicine, Pusan National University, Yangsan 50612, Republic of Korea

**Keywords:** butyl benzyl phthalate, aging, seminal vesicle, oxidative stress, FOXO signaling

## Abstract

Butyl benzyl phthalate (BBP), a widely used endocrine-disrupting chemical, has been associated with reproductive toxicity; however, its long-term effects during aging remain poorly understood. In the present study, we examined the effects of prolonged BBP exposure on the male reproductive system using naturally aged C57BL/6J mice. Mice received BBP at 169 μg/kg/day in drinking water for 10 or 22 months and were analyzed at 24 months of age. No significant differences in body weight, food intake, or water consumption were observed among the experimental groups. Representative gross images showed apparent distension and dark-red discoloration of the seminal vesicles in BBP-exposed aged mice. Compared with Young mice, the BBP-exposed aged groups showed higher expression of IL-1β, IL-6, TNFα, SOD1, and SOD2 and lower CAT expression in the seminal vesicle. H_2_DCFDA fluorescence showed a non-significant increasing trend. Because an untreated age-matched Old group was not included in the seminal vesicle analyses, the effects of aging and BBP could not be distinguished. In contrast, the testis showed limited changes in inflammatory cytokine-, antioxidant enzyme-, and steroidogenesis-related gene expression and in H_2_DCFDA fluorescence relative to the untreated Old group. Exploratory miRNA sequencing of pooled testicular RNA and comparison with a TM3 Leydig cell dataset identified miR-664-5p as a candidate showing higher relative abundance in both datasets. TargetScan analysis predicted binding sites for miR-664-5p in FOXO1 and FOXO3. In BBP-treated TM3 cells, FOXO3 protein expression was decreased, and FOXO6 expression was increased, whereas FOXO1 showed no consistent dose-dependent change. These molecular findings do not establish direct regulation of FOXO proteins by miR-664-5p or explain the seminal vesicle findings.

## 1. Introduction

Aging is accompanied by a progressive decline in tissue homeostasis, leading to impaired organ function and increased susceptibility to environmental stress [1,2]. The male reproductive system undergoes progressive functional decline with aging, as normal reproductive function depends on the coordinated activity of the testis and accessory reproductive glands [3]. In addition to intrinsic aging, humans are continuously exposed to a wide variety of environmental chemicals throughout life [4,5]. Despite this lifelong exposure, most experimental studies investigating reproductive toxicity have relied on young animals exposed for relatively short periods. As a result, the biological effects of prolonged environmental exposure during aging remain poorly understood [6].

Endocrine-disrupting chemicals (EDCs) interfere with hormone signaling and have been associated with reproductive disorders as well as metabolic and age-related diseases [7,8]. Among them, butyl benzyl phthalate (BBP) is widely used in plastics and many consumer products, resulting in continuous human exposure [9]. Previous studies have shown that BBP and other phthalates induce oxidative stress, inflammatory responses, mitochondrial dysfunction, and epigenetic alterations in multiple tissues [5,10,11,12,13]. Our previous study demonstrated that the biological effects of BBP differ according to age, promoting hepatic steatosis in young mice while enhancing fibrosis in aged mice [14]. These findings suggest that tissue responses to chronic BBP exposure may differ across organs and age groups. However, whether similar age-dependent effects occur in the male reproductive system remains unknown.

Although the testis has been the primary focus of studies examining phthalate-induced reproductive toxicity, considerably less attention has been given to accessory reproductive glands. Among these, the seminal vesicle plays an essential role in male fertility by producing most of the seminal plasma and secreting proteins required for sperm survival and fertilization [15]. Because its development and secretory function are tightly regulated by androgen signaling, the seminal vesicle may be particularly susceptible to endocrine-disrupting chemicals with anti-androgenic activity [10,11]. In contrast, the testis serves as the primary endocrine organ of the male reproductive system and is responsible for both steroid hormone production and spermatogenesis [16,17]. Whether prolonged BBP exposure affects these reproductive organs differently during aging remains largely unknown.

Recent studies have shown that environmental exposure can alter gene expression and microRNA profiles before substantial tissue remodeling becomes evident [18,19]. Among the molecular mechanisms involved in cellular adaptation to environmental stress, microRNAs have emerged as important post-transcriptional regulators that respond to diverse environmental stimuli [19,20,21]. Their downstream target genes participate in multiple biological processes, including oxidative stress responses, apoptosis, inflammation, and cellular homeostasis [22]. However, the contribution of miRNA-mediated regulatory pathways to long-term BBP-induced reproductive toxicity during aging remains unclear.

In the present study, we examined the effects of long-term BBP exposure on the seminal vesicle and testis in aged mice. We also performed exploratory miRNA sequencing in testicular tissue and compared the results with those obtained from BBP-treated TM3 Leydig cells to identify candidate molecular responses. These analyses were not intended to define a mechanism underlying the seminal vesicle findings.

## 2. Materials and Methods

### 2.1. Mice and BBP Exposure

Mice were allocated to experimental groups by simple random allocation before the start of BBP exposure. All mice were maintained under identical specific pathogen-free housing conditions, including temperature, humidity, light/dark cycle, chow, and water access. Body weight, food intake, and water consumption were measured monthly in all groups, and the BBP concentration in drinking water was adjusted according to body weight and water intake to maintain the target dose. Cage location and measurement order were not specifically randomized.

Butyl benzyl phthalate (BBP; Sigma-Aldrich, St. Louis, MO, USA) was administered via drinking water at a dose of 169 μg/kg/day. Mice in the Old + BBP (10 months) group received BBP from 14 to 24 months of age, whereas mice in the Old + BBP (22 months) group received BBP from 2 to 24 months of age. All aged mice, including the Old, Old + BBP (10 months), and Old + BBP (22 months) groups, were sacrificed at 24 months of age.

Each experimental group initially consisted of six mice. The Young group was analyzed at 6 months of age, whereas the Old, Old + BBP (10 months), and Old + BBP (22 months) groups were analyzed at 24 months of age. During the long-term study, several mice in the aged groups died before the scheduled 24-month endpoint. Consequently, the final numbers of animals differed among the aged groups. At the endpoint, the Young, Old, Old + BBP (10 months), and Old + BBP (22 months) groups included *n* = 6, *n* = 4, *n* = 5, and *n* = 4 mice, respectively. The sample sizes reported in the manuscript represent the numbers of mice that reached the scheduled endpoint and were available for tissue collection and subsequent analyses. Animals that died before the endpoint were not included in the endpoint analyses, and missing values were not imputed.

Body weight, food intake, and water consumption were monitored monthly throughout the experiment. The concentration of BBP in the drinking water was adjusted according to body weight and water intake to maintain the target dose. At the end of the experimental period, mice were euthanized, and blood, testes, and seminal vesicles were collected. The tissues were immediately snap-frozen in liquid nitrogen and stored at −80 °C until further analysis.

All animal procedures were approved by the Institutional Animal Care and Use Committee (IACUC) of Kyungsung University (Approval No. Research-21-020B; approval date: 26 January 2023) and were performed in accordance with the relevant institutional guidelines. This study was conducted and reported in accordance with the ARRIVE guidelines. Mice with severe fighting-related wounds or injuries were excluded for animal welfare reasons and to avoid potential confounding effects of trauma, inflammation, or stress.

### 2.2. Quantitative Reverse Transcriptase Polymerase Chain Reaction (qRT-PCR)

Total RNA was extracted from the testes, seminal vesicles, and TM3 cells using the Total RNA Extraction Kit (SMART GENE, Daejeon, Republic of Korea) according to the manufacturer’s instructions. RNA concentration and purity were assessed using a Nano-300 spectrophotometer (Allsheng, Hangzhou, Zhejiang, China). Complementary DNA (cDNA) was synthesized from 400 ng of total RNA using the Compact cDNA Synthesis Kit (SMART GENE, Daejeon, Republic of Korea). Gene expression levels were quantified by quantitative reverse transcriptase polymerase chain reaction (qRT-PCR) using the SYBR Green Master Mix (SMART GENE, Daejeon, Republic of Korea) on a QuantStudio 1 Real-Time PCR System (Thermo Fisher Scientific, Waltham, MA, USA). Primers specific for genes related to oxidative stress, antioxidant defense, steroidogenesis, fibrosis, and inflammation were used and are listed in Supplementary Table S1. Gene expression levels were normalized to 18S as the internal reference gene, and relative expression levels were calculated using the 2^−ΔΔCt^ method.

### 2.3. Measurement of Reactive Oxygen Species (ROS)

Reactive oxygen species-associated fluorescence in mouse seminal vesicle and testicular tissues was measured using 2′,7′-dichlorodihydrofluorescein diacetate (H_2_DCFDA; Invitrogen, Carlsbad, CA, USA). Frozen tissues were homogenized in ice-cold phosphate-buffered saline (PBS), and protein concentrations were determined using the Bradford protein assay. To generate the fluorescent probe, H_2_DCFDA (12.5 mM) was incubated with esterase (600 U) at 30 °C for 20 min before use. Equal amounts of protein were incubated with 100 μM H_2_DCFDA at 37 °C for 1 h in the dark. Fluorescence intensity was measured using a SpectraMax iD3 multimode microplate reader (Molecular Devices, San Jose, CA, USA) at excitation and emission wavelengths of 485 and 535 nm, respectively. Each tissue sample obtained from one mouse was considered one biological replicate. Measurements were performed in technical duplicate, and the mean of the technical measurements was normalized to the protein concentration of the corresponding sample. The averaged value was used as one biological observation for statistical analysis.

### 2.4. TM3 Cell Culture and BBP Treatment

TM3 mouse Leydig cells were obtained from the Korean Cell Line Bank (KCLB, Seoul, Republic of Korea) and maintained in a 1:1 (*v*/*v*) mixture of Dulbecco’s Modified Eagle’s Medium and DMEM/Nutrient Mixture F-12 (DMEM/F-12; Gibco, Thermo Fisher Scientific, Waltham, MA, USA), supplemented with 5% horse serum, 2.5% FBS and 100 U/mL penicillin at 37 °C in a humidified atmosphere containing 5% CO_2_. Cells were seeded at 5 × 10^5^ cells/well and treated at approximately 80% confluence. BBP was dissolved in DMSO and added to the culture medium at final concentrations of 5 or 50 μM. Vehicle-treated control cells received the same final concentration of 0.1% DMSO. Cells were exposed to BBP for 24 h before protein collection. Each independently conducted cell culture and BBP treatment was considered one biological replicate.

### 2.5. Western Blot Analysis

Protein expression levels of FOXO family members in TM3 Leydig cells were analyzed by Western blot analysis. TM3 Leydig cells were lysed in RIPA buffer supplemented with protease inhibitors. Protein concentrations were determined using the Pierce™ BCA Protein Assay Kit (Thermo Fisher Scientific, Waltham, MA, USA). Equal amounts of protein (20 μg) were separated on 10% SDS-PAGE gels and electrophoretically transferred onto PVDF membranes (Merck Millipore, Burlington, MA, USA). Membranes were blocked with 5% non-fat dry milk in Tris-buffered saline containing 0.1% Tween-20 (TBST) for 1 h at room temperature and incubated overnight at 4 °C with primary antibodies against FOXO1 (1:1000, Abclonal, Wuhan, China), FOXO3 (1:1000, Santa Cruz Biotechnology, Dallas, TX, USA), FOXO6 (1:1000, Proteintech, Rosemont, IL, USA), and β-actin (1:1000, Abclonal, Wuhan, China). After washing with TBST, membranes were incubated with IRDye^®^ 680RD Goat anti-Rabbit IgG (1:10,000 dilution; LI-COR, Lincoln, NE, USA) for 1 h 30 min at room temperature with gentle shaking. Protein bands were visualized using the Odyssey^®^ XF Imaging System (LI-COR), and band intensities were quantified using Empiria studio (v2.3; LI-COR) and normalized to β-actin.

### 2.6. Exploratory miRNA Sequencing and Target Prediction Analysis

miRNA sequencing was performed as an exploratory analysis to identify candidate miRNAs associated with BBP exposure in aged mouse testes and TM3 Leydig cells. For testicular miRNA sequencing, total RNA was extracted individually from the testes of three untreated aged mice and three BBP-exposed aged mice. RNA concentration and purity were assessed using a Nano-300 spectrophotometer (Allsheng, Hangzhou, China). Equal amounts of RNA from the three mice in each group were pooled to generate one sequencing library per group.

Small RNA libraries were prepared using [kit name and manufacturer] according to the manufacturer’s instructions and sequenced using an Illumina sequencing platform. Raw reads were processed by removing adapter sequences and low-quality reads. Clean reads were aligned to known mouse miRNAs in miRBase, and miRNA abundance was normalized as counts per million mapped reads (CPM).

Because one pooled testicular RNA library was generated per group, inter-individual variability could not be assessed, and no statistical differential expression analysis was performed. The criteria of |log_2_FC| ≥ 1 and CPM ≥ 10 were used only as descriptive filtering criteria to select candidate miRNAs showing differences in relative abundance between pooled libraries. No *p* values or false-discovery rate corrections were calculated for the testicular sequencing data.

Candidate miRNAs identified in the pooled testicular libraries were compared with those identified in BBP-treated TM3 Leydig cells. Target genes of selected candidate miRNAs were predicted using TargetScanMouse. TargetScan predictions were used to select proteins for subsequent exploratory evaluation and were not considered evidence of direct miRNA–target regulation.

### 2.7. Statistical Analyses

Statistical analyses were performed using GraphPad Prism 11 (GraphPad Software, San Diego, CA, USA). Data are presented as the mean ± standard error of the mean (SEM). Normality was assessed using the Shapiro–Wilk test, and homogeneity of variance was assessed using the Brown–Forsythe test. Differences among multiple groups were analyzed using one-way analysis of variance (ANOVA) followed by Dunnett’s multiple comparisons test. If the assumptions for parametric analysis were not met, non-parametric analyses were considered. A value of *p* < 0.05 was considered statistically significant. Group allocation was known during animal allocation and BBP administration, but investigators responsible for outcome assessment and data analysis were blinded to group allocation until the analyses were completed.

## 3. Results

### 3.1. Physiological Measures and Gross Seminal Vesicle Findings in BBP-Exposed Aged Mice

**Figure 1:**
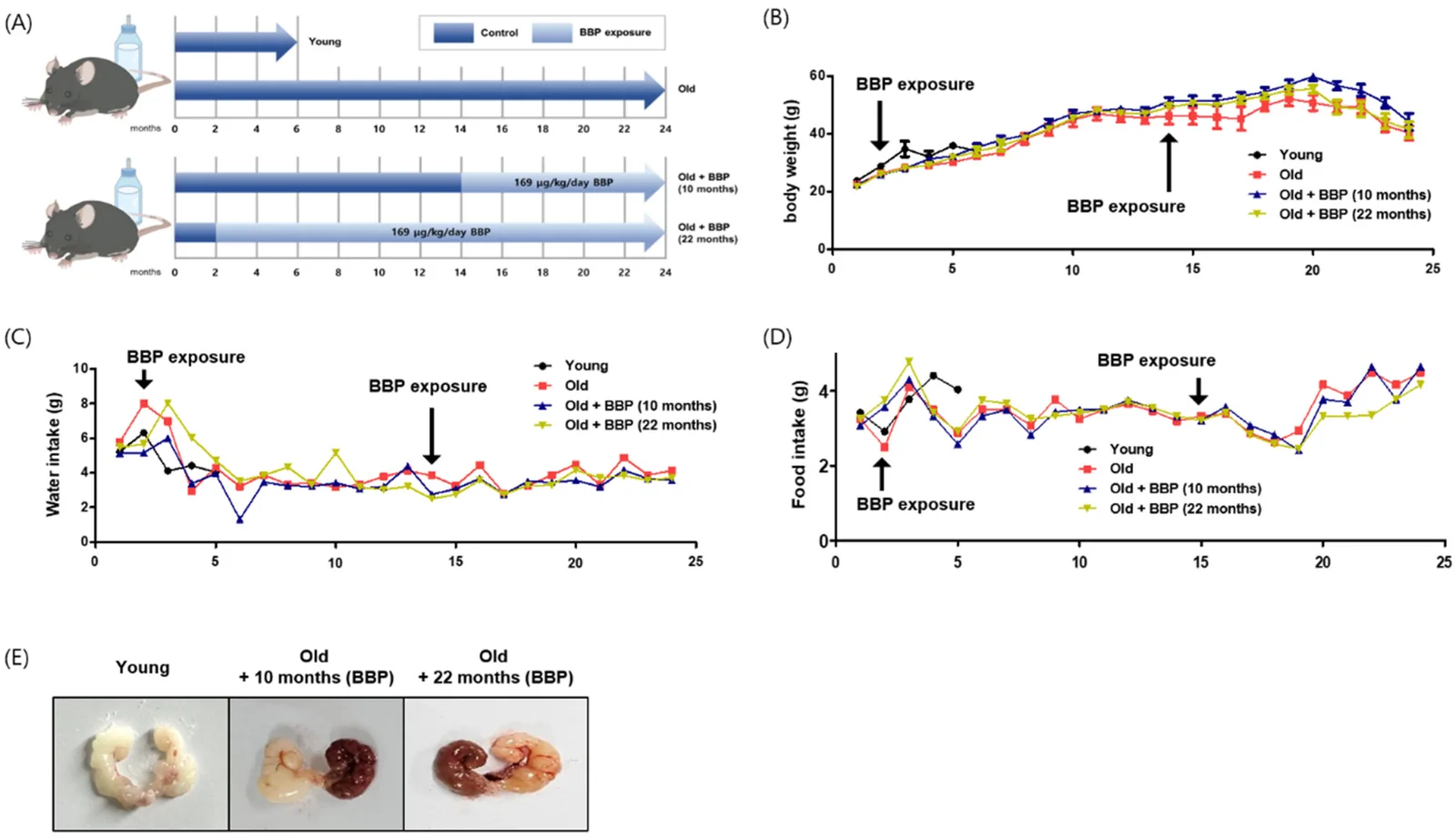
Experimental design, physiological measures, and gross seminal vesicle findings. (**A**) Experimental design. Male C57BL/6J mice were assigned to the Young, Old, Old + BBP (10 months), and Old + BBP (22 months) groups. Young mice were analyzed at 6 months of age. Old mice were maintained without BBP exposure and analyzed at 24 months of age. Mice in the Old + BBP (10 months) group received BBP at 169 μg/kg/day from 14 to 24 months of age, whereas mice in the Old + BBP (22 months) group received BBP from 2 to 24 months of age. Monthly body weight (**B**), water intake (**C**), and food intake (**D**) were measured during the experimental period. Exact group sizes were Young, *n* = 6; Old, *n* = 4; Old + BBP (10 months), *n* = 5; and Old + BBP (22 months), *n* = 4. (**E**) Representative gross images of seminal vesicles from Young, Old + BBP (10 months), and Old + BBP (22 months) mice. Apparent distension and dark-red discoloration were observed in the BBP-exposed aged groups. Seminal vesicle wet weight and histological changes were not assessed.

To investigate the effects of long-term BBP exposure during aging, mice received BBP at 169 μg/kg/day in drinking water for 10 or 22 months (Figure 1A). Monthly body weight, water intake, and food intake are shown in Figure 1B–D, respectively. No significant differences in these parameters were observed among the experimental groups.

Representative gross images showed apparent distension and dark-red discoloration of the seminal vesicles in BBP-exposed aged mice (Figure 1E). Seminal vesicle wet weight and histological changes were not assessed. In addition, an untreated age-matched Old group was not included in the seminal vesicle assessment. Therefore, quantitative enlargement, histologically confirmed hemorrhage, and BBP-specific morphological effects could not be established.

### 3.2. Inflammatory Cytokine- and Antioxidant-Related Gene Expression in the Seminal Vesicle

**Figure 2:**
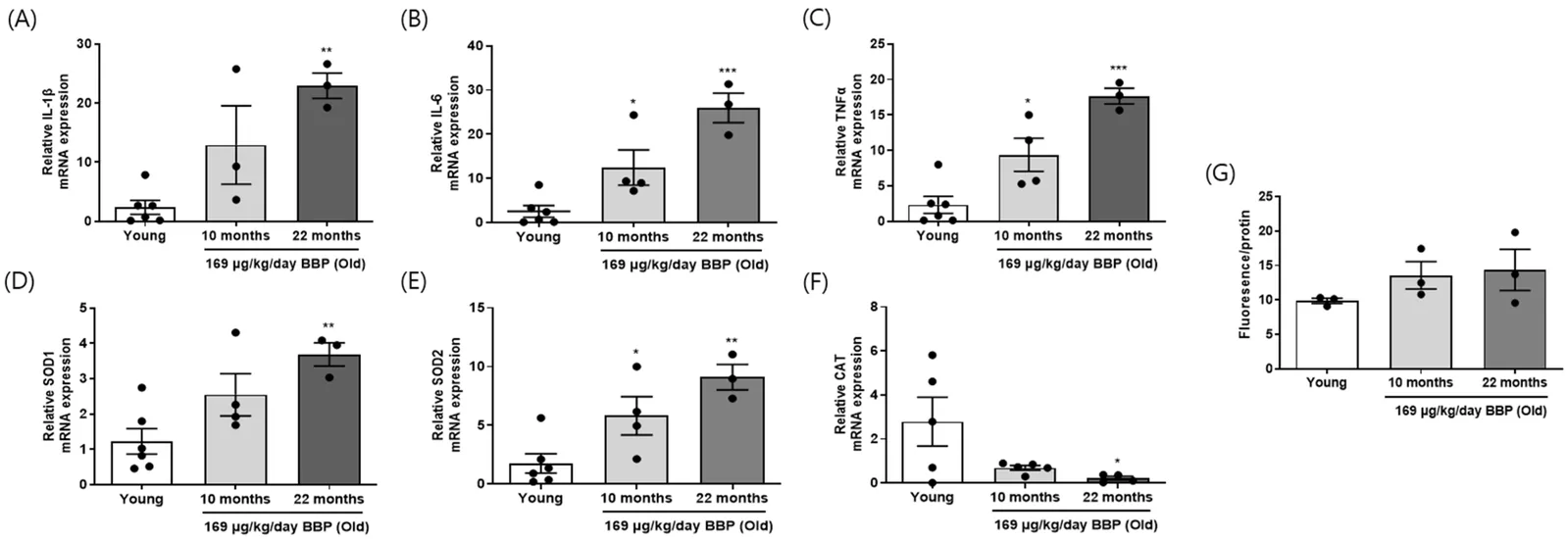
Inflammatory cytokine- and antioxidant-related gene expression in the seminal vesicle. Relative mRNA expression levels of IL-1β (**A**), IL-6 (**B**), TNFα (**C**), SOD1 (**D**), SOD2 (**E**), and CAT (**F**) in seminal vesicle tissues from Young and BBP-exposed aged mice. (**G**) H_2_DCFDA fluorescence normalized to protein concentration in seminal vesicle homogenates. Data are presented as individual biological observations with the mean ± SEM. Each point represents one mouse. Sample sizes varied among qRT-PCR targets because of sample availability and quality control; the exact *n* for each analysis is provided in Supplementary Table S2. For the H_2_DCFDA assay, *n* = 3 per group. Statistical comparisons were performed using one-way ANOVA followed by Dunnett’s multiple-comparisons test, with comparisons made against the Young group (* *p* < 0.05, ** *p* < 0.01, and *** *p* < 0.001). Exact adjusted *p* values, effect sizes, and 95% confidence intervals are provided in Supplementary Table S2. An untreated age-matched Old group was not included in this analysis.

To examine inflammatory cytokine-related gene expression in the seminal vesicle, IL-1β, IL-6, and TNFα mRNA levels were measured in Young and BBP-exposed aged mice. The expression levels of all three cytokines were higher in the BBP-exposed aged groups than in the Young group (Figure 2A–C).

The expression levels of antioxidant-related genes were also examined. SOD1 and SOD2 mRNA expression was higher, whereas CAT expression was lower, in the BBP-exposed aged groups than in the Young group (Figure 2D–F).

H_2_DCFDA fluorescence in seminal vesicle homogenates showed a non-significant increasing trend (Figure 2G). These results show altered inflammatory cytokine- and antioxidant-related gene expression but do not establish increased ROS or oxidative stress. Because an untreated age-matched Old group was not included in this analysis, the effects of aging and BBP could not be distinguished.

### 3.3. Inflammatory and Antioxidant-Related Responses in the Testis

**Figure 3:**
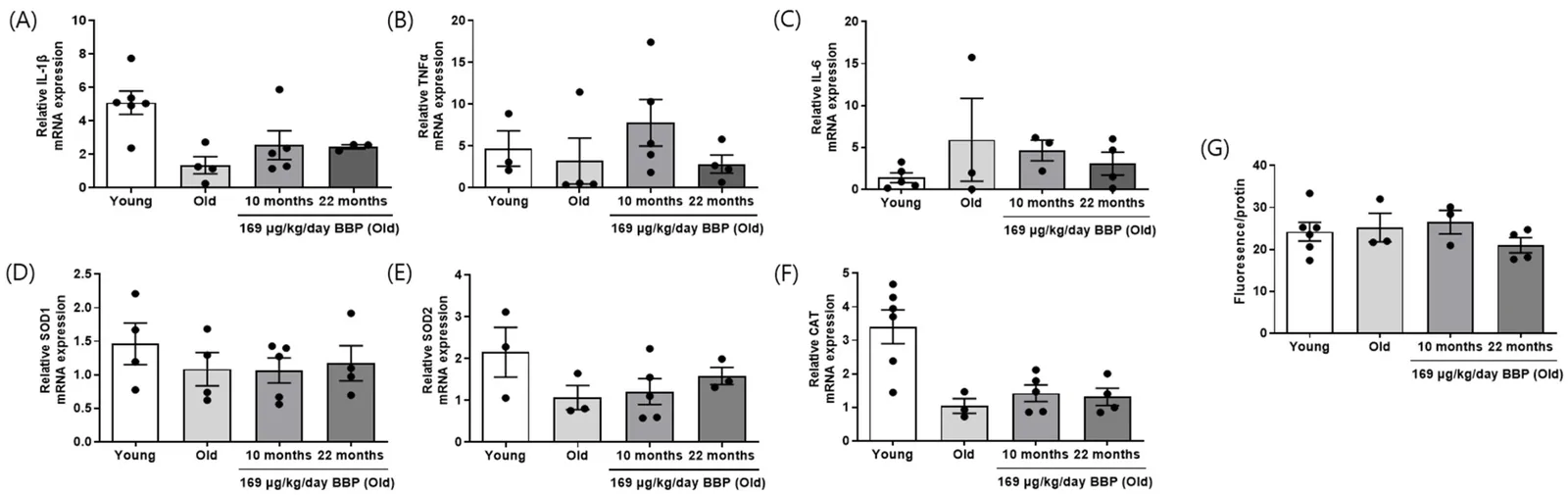
Inflammatory and antioxidant-related responses in the testis. Relative mRNA expression levels of IL-1β (**A**), TNFα (**B**), IL-6 (**C**), SOD1 (**D**), SOD2 (**E**), and CAT (**F**) in testicular tissues from Young, Old, Old + BBP (10 months), and Old + BBP (22 months) mice. (**G**) H_2_DCFDA fluorescence in testicular homogenates. Data are presented as individual biological observations with the mean ± SEM. Each point represents one mouse. Sample sizes varied among qRT-PCR targets because of sample availability and quality control; the exact *n* for each analysis is provided in Supplementary Table S3. For the H_2_DCFDA assay were *n* = 6, 3, 3, and 4, respectively. Statistical comparisons were performed using one-way ANOVA followed by Dunnett’s multiple-comparisons test, with comparisons made against the Old group. Exact adjusted *p* values, effect sizes, and 95% confidence intervals are provided in Supplementary Table S3.

To determine whether long-term BBP exposure was associated with inflammatory changes in the testis, IL-1β, IL-6, and TNFα mRNA expression was examined. No significant differences in these cytokines were observed between the untreated Old group and the BBP-exposed aged groups, although TNFα showed a non-significant increasing trend after 10 months of BBP exposure (Figure 3A–C).

SOD1, SOD2, and CAT mRNA expression also did not differ significantly between the untreated Old group and the BBP-exposed aged groups (Figure 3D–F). H_2_DCFDA fluorescence in testicular homogenates showed no significant differences among the aged groups (Figure 3G). These results indicate that the testis showed limited changes in the inflammatory and antioxidant-related endpoints examined.

### 3.4. Exploratory Screening of Candidate miRNAs Associated with BBP Exposure

**Figure 4:**
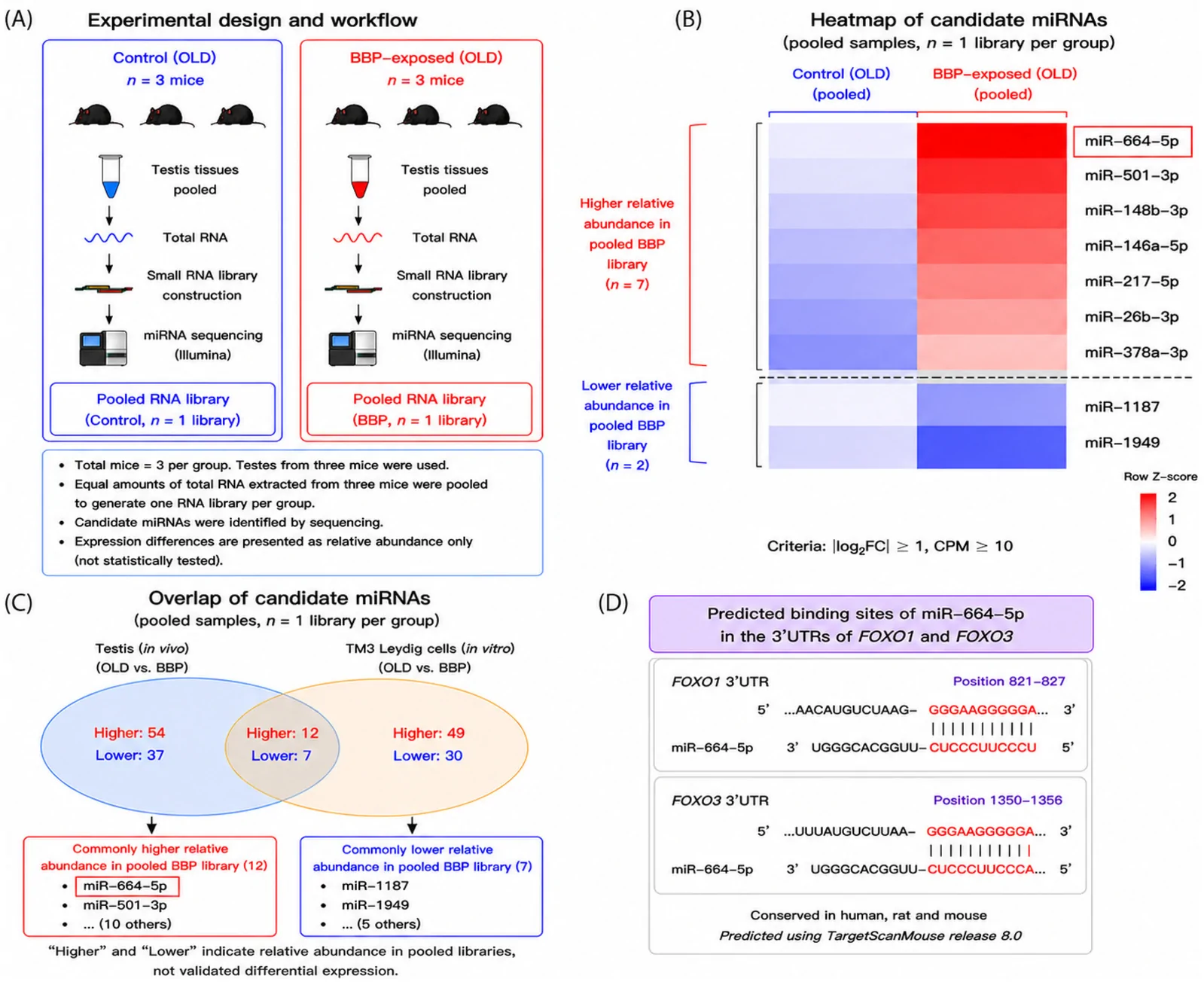
Exploratory screening of candidate miRNAs associated with BBP exposure. (**A**) Experimental workflow for exploratory miRNA sequencing. Total RNA was extracted individually from the testes of three untreated aged mice and three BBP-exposed aged mice. Equal amounts of RNA were pooled to generate one sequencing library per group. Because biological replicate libraries were not generated, no statistical differential expression analysis was performed. (**B**) Heatmap showing descriptive differences in relative miRNA abundance between the pooled testicular libraries. The criteria of |log_2_FC| ≥ 1 and CPM ≥ 10 were used only for descriptive candidate filtering. (**C**) Venn diagram comparing candidate miRNAs showing concordant directional differences in the pooled testicular and TM3 Leydig cell datasets. miR-664-5p showed higher relative abundance in both datasets. (**D**) TargetScanMouse prediction of binding sites for miR-664-5p in the 3′UTRs of FOXO1 and FOXO3.

Exploratory small RNA sequencing was performed using pooled testicular RNA from untreated aged and BBP-exposed aged mice (Figure 4A). Equal amounts of RNA from three mice were pooled to generate one sequencing library per group. Because biological replicate libraries were not generated, the sequencing data were analyzed descriptively, and no statistical differential expression analysis was performed.

Using |log_2_FC| ≥ 1 and CPM ≥ 10 as descriptive filtering criteria, seven candidate miRNAs showed higher relative abundance and two showed lower relative abundance in the pooled BBP-exposed testicular library compared with the pooled aged-control library (Figure 4B).

The testicular sequencing results were compared with those obtained from BBP-treated TM3 Leydig cells. miR-664-5p was selected as a candidate because it showed higher relative abundance in both datasets (Figure 4C). TargetScan analysis predicted binding sites for miR-664-5p in the 3′UTRs of FOXO1 and FOXO3 (Figure 4D). These predictions provided the rationale for examining FOXO protein expression in TM3 cells but did not demonstrate direct regulation.

### 3.5. FOXO Family Protein Expression in BBP-Treated TM3 Leydig Cells

**Figure 5:**
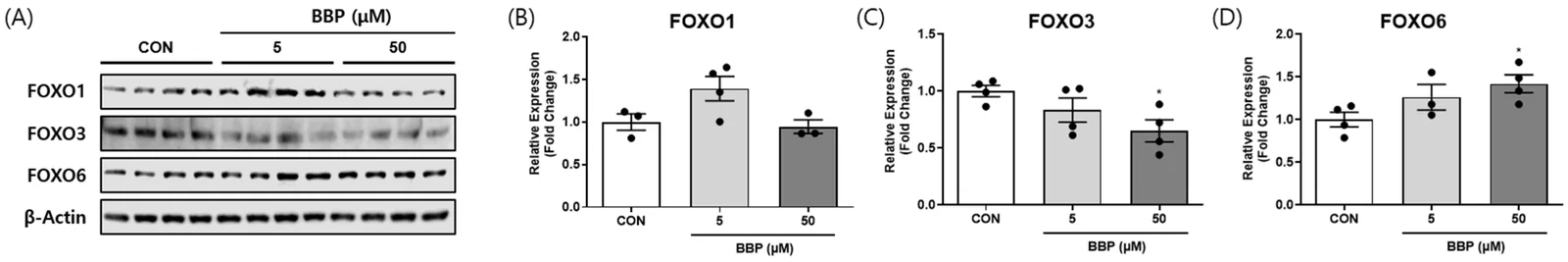
FOXO family protein expression in BBP-treated TM3 Leydig cells. (**A**) Representative Western blot images of FOXO1, FOXO3, and FOXO6 in TM3 Leydig cells treated with vehicle, 5 μM BBP, or 50 μM BBP for 24 h. β-Actin was used as the loading control. Densitometric quantification of FOXO1 (**B**), FOXO3 (**C**), and FOXO6 (**D**) expression normalized to β-actin. Data are presented as individual biological observations with the mean ± SEM. Each point represents one independently conducted cell culture and treatment experiment. The exact *n* for each analysis is provided in Supplementary Table S4. Statistical comparisons were performed using one-way ANOVA followed by Dunnett’s multiple-comparisons test, with the vehicle-control group used as the reference (* *p* < 0.05). Exact adjusted *p* values, effect sizes, and 95% confidence intervals are provided in Supplementary Table S4.

Based on the TargetScan prediction of FOXO1 and FOXO3 as potential targets of miR-664-5p, their protein expression was examined in BBP-treated TM3 Leydig cells (Figure 5A). FOXO6 was included as an additional FOXO family member and was not predicted as a miR-664-5p target.

FOXO1 protein expression was higher following treatment with 5 μM BBP than in vehicle-treated control cells but was comparable to the control level following treatment with 50 μM BBP (Figure 5B). FOXO3 protein expression was lower following treatment with 50 μM BBP, whereas FOXO6 expression was higher at the same concentration (Figure 5C,D). Exact adjusted *p* values are provided in Supplementary Table S4.

Because miR-664-5p was not experimentally manipulated and direct binding was not examined, these findings do not demonstrate that the FOXO protein changes were mediated by miR-664-5p.

## 4. Discussion

In the present study, we investigated the effects of long-term BBP exposure on the aging male reproductive system. Humans are continuously exposed to endocrine-disrupting chemicals (EDCs) throughout life [7,8]; however, most experimental studies have evaluated their biological effects using relatively short-term exposure models in young animals [6]. As a result, the biological consequences of prolonged exposure during aging remain poorly understood. To better reflect chronic environmental exposure over the lifespan, mice were exposed to BBP for up to 22 months and analyzed at 24 months of age. Body weight, food intake, and water consumption did not differ among the experimental groups. However, these measurements do not exclude systemic effects that were not evaluated in the present study.

The seminal vesicle is a major accessory sex gland that produces most components of seminal plasma and secretes proteins and bioactive factors required for normal sperm function [15]. Its development and secretory activity are strongly regulated by androgen signaling [15], which may contribute to its sensitivity to endocrine disruptors with anti-androgenic activity, including BBP [10,11]. Previous studies have reported phthalate-associated changes in the seminal vesicle [12,23]. In the present study, gross images of BBP-exposed aged mice showed apparent distension and dark-red discoloration of the seminal vesicle. However, an untreated age-matched Old group was not included in the seminal vesicle analyses, and seminal vesicle weight and histological changes were not assessed. Therefore, the effects of aging and BBP could not be distinguished, and quantitative enlargement, hemorrhage, or tissue injury could not be confirmed. Rather than providing the first evidence of seminal vesicle inflammation, the present study extends previous observations to a prolonged exposure model using naturally aged mice.

Oxidative stress is considered an important mechanism of phthalate-induced reproductive toxicity [12,24]. In the seminal vesicle of BBP-exposed aged mice, SOD1 and SOD2 mRNA expression was higher, whereas CAT expression was lower. Because superoxide dismutases convert superoxide radicals into hydrogen peroxide and catalase contributes to hydrogen peroxide removal [25], this expression pattern may reflect altered or compensatory regulation of antioxidant-related genes. However, H_2_DCFDA fluorescence showed only a non-significant increasing trend, and lipid peroxidation, glutathione status, protein oxidation, and oxidative DNA damage were not examined. Therefore, the present findings do not establish oxidative stress or oxidative tissue damage.

In contrast to the seminal vesicle findings, the testis showed limited changes in the inflammatory cytokine-, antioxidant enzyme-, steroidogenesis-related, and ROS endpoints examined. No significant differences in these endpoints were observed between the untreated Old group and the BBP-exposed aged groups. The present data therefore indicate different response patterns in the seminal vesicle and testis but do not establish that the testis is generally resistant to prolonged BBP exposure.

The different response patterns may be related to differences in androgen dependence, secretory activity, cellular composition, local metabolism, and tissue organization. The specialized microenvironment and blood–testis barrier may also influence the testicular response [26]. In particular, seminal vesicle growth and secretory function are strongly regulated by androgen receptor signaling [15]. However, serum or intratesticular testosterone, gonadotropins, androgen receptor expression, and androgen receptor activity were not examined. Therefore, the contribution of endocrine changes to the seminal vesicle findings remains unknown.

Although the testis showed limited changes in the inflammatory and antioxidant-related endpoints examined, this did not exclude other molecular responses. Testicular miRNA profiling was therefore performed as an exploratory analysis and compared with data obtained from BBP-treated TM3 Leydig cells. The testis was selected because it is a major target tissue in studies of phthalate-induced reproductive toxicity and allowed comparison with a Leydig cell model [27,28]. However, testicular tissue was not used as a surrogate for the seminal vesicle.

Exploratory comparison of the testicular and TM3 datasets identified miR-664-5p as a candidate showing higher relative abundance in both datasets. Because equal amounts of RNA from three mice were pooled to generate one testicular sequencing library per group, inter-individual variability could not be assessed and statistical differential expression analysis was not possible [29,30]. The observed difference in miR-664-5p abundance therefore requires validation using individual biological samples. TargetScan analysis predicted binding sites for miR-664-5p in the 3′ untranslated regions of FOXO1 and FOXO3, which provided the rationale for examining FOXO protein expression in TM3 cells [31].

FOXO transcription factors are involved in the regulation of cellular metabolism, stress responses, and aging [32,33]. FOXO3 also contributes to antioxidant gene regulation [32,34,35]. In the present study, FOXO3 protein expression was lower following treatment with 50 μM BBP, whereas FOXO6 expression was higher. FOXO1 did not show a consistent dose-dependent change. However, the concurrent difference in miR-664-5p abundance and FOXO3 protein expression does not demonstrate direct regulation. miR-664-5p was not experimentally manipulated, and direct binding to FOXO1 or FOXO3 was not examined. FOXO6 was not identified as a predicted miR-664-5p target, and the significance of its increased expression remains unclear.

**Figure 6:**
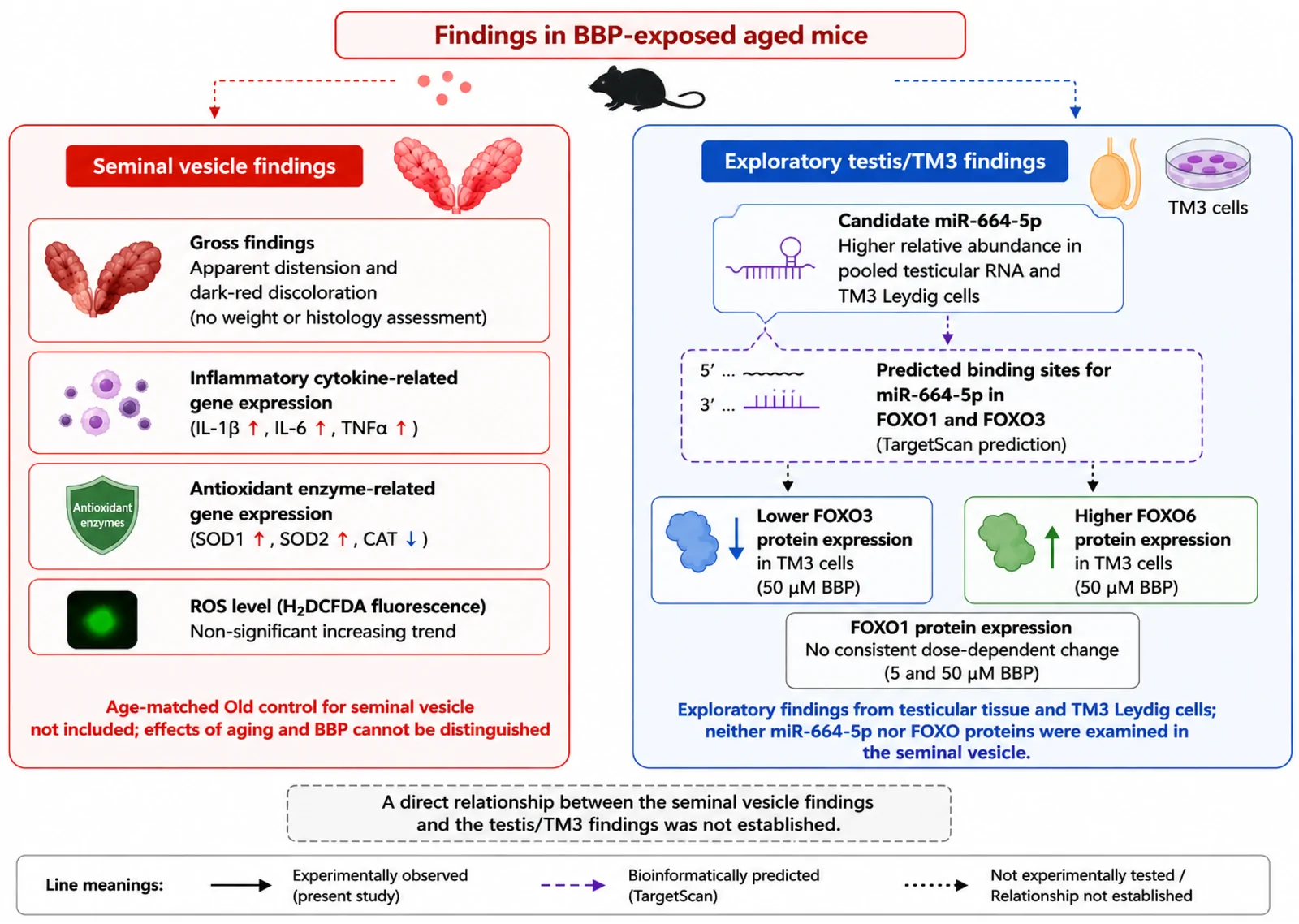
Summary of findings associated with long-term BBP exposure. Gross seminal vesicle findings and changes in inflammatory cytokine- and antioxidant-related gene expression are shown separately from the exploratory miRNA and FOXO findings obtained from testicular tissue and TM3 Leydig cells. miR-664-5p showed higher relative abundance in the pooled testicular library and the TM3 cell dataset. TargetScan predicted binding sites for miR-664-5p in FOXO1 and FOXO3. In BBP-treated TM3 cells, FOXO3 protein expression was lower and FOXO6 expression was higher, whereas FOXO1 showed no consistent dose-dependent change. Solid lines indicate findings observed in the present study, dashed lines indicate TargetScan predictions, and dotted lines indicate relationships that were not experimentally tested. A direct link between the seminal vesicle findings and the testis/TM3 findings was not established.

Neither miR-664-5p nor FOXO proteins were examined in the seminal vesicle. Therefore, the miRNA and FOXO findings obtained from testicular tissue and TM3 Leydig cells cannot explain the seminal vesicle findings observed in this study. Figure 6 summarizes the seminal vesicle findings separately from the exploratory testis and TM3 findings. A direct relationship between these findings was not established.

Several limitations should be considered. An untreated age-matched Old group was not included in the seminal vesicle analyses, and seminal vesicle wet weight and histopathological changes were not assessed. Therefore, the effects of aging and BBP could not be distinguished, and the gross findings could not be confirmed quantitatively or histologically. H_2_DCFDA fluorescence also showed only a non-significant increasing trend, and additional markers of oxidative stress or oxidative damage were not examined. In addition, testosterone, gonadotropins, and androgen receptor signaling were not measured.

The miRNA sequencing analysis was based on one pooled testicular library per group, which prevented assessment of biological variability and statistical differential expression. Neither miR-664-5p nor FOXO proteins were examined in the seminal vesicle, and direct regulation of FOXO1 or FOXO3 by miR-664-5p was not tested. These limitations prevent a mechanistic link from being established between the seminal vesicle findings and the molecular changes observed in the testis and TM3 cells. The prostate and other accessory reproductive glands were also not examined.

In conclusion, BBP-exposed aged mice showed gross seminal vesicle abnormalities together with altered inflammatory cytokine- and antioxidant-related gene expression. However, the absence of an untreated age-matched Old group, seminal vesicle wet weight measurements, and histological analysis limits the interpretation of these findings. The testis showed limited changes in the endpoints examined. Exploratory analyses also identified miR-664-5p as a candidate showing higher relative abundance in pooled testicular RNA and TM3 Leydig cells and showed altered FOXO protein expression in BBP-treated TM3 cells. These molecular findings do not establish direct miR-664-5p–FOXO regulation or explain the seminal vesicle findings.

## Data Availability

The data presented in this study are available on request from the corresponding author.

## Supplementary Materials

The following supporting information can be downloaded at: https://www.mdpi.com/article/10.3390/antiox15081021/s1, Table S1: Primer sequences used in qRT-PCR analysis; Table S2: Detailed statistical parameters for quantitative outcomes in the seminal vesicle; Table S3: Detailed statistical parameters for quantitative outcomes in the testis; Table S4: Detailed statistical parameters for quantitative outcomes in TM3 Leydig cell experiments.
