# Supplementary material for: Seminal Vesicle Abnormalities and Exploratory miR-664-5p and FOXO Findings in Aged Mice Following Long-Term Butyl Benzyl Phthalate Exposure
Source: Antioxidants (Basel). 2026 Aug 17;15(8):1021. doi: 10.3390/antiox15081021 (PMC13509467; doi:10.3390/antiox15081021)
Supplement: Supplementary file 1 [file antioxidants-15-01021-s001.zip › antioxidants-4433495-supplementary.pdf]

Supplementary Table S1. Primer sequences used in qRT-PCR analysis.

Gene	Forward primer sequence (5′ → 3′)	Reverse primer sequence (5′ → 3′)
18S	GTAACCCGTTGAACCCCATT	CCATCCAATCGGTAGTAGCG
IL6	GCAGCATCACCTTCGCTTAGA	CAGATATTGGCATGGGAGCAAG
IL1β	GCAACTGTTCCTGAACTCAACT	ATCTTTTGGGGTCCGTCAACT
TNFα	CAGGCGGTGCCTATGTCTC	CGATCACCCCGAAGTTCAGTAG
SOD1	AACCAGTTGTGTTGTCAGGAC	CCACCATGTTTCTTAGAGTGAGG
SOD2	CAGACCTGCCTTACGACTATGG	CTCGGTGGCGTTGAGATTGTT
CAT	AGCGACCAGATGAAGCAGTG	TCCGCTCTCTGTCAAAGTGTG

Supplementary Table S2. Detailed statistical parameters for quantitative outcomes in the seminal vesicle.

Figure	Outcome	Groups, *n*	ANOVA	*P*-value	η²	Post hoc comparison	Mean difference	95% CI	Adjusted *P*
Fig. 2A	IL-1β	Young, n=6; 10 months, n=3; 22 months, n=3	F(2,9)=11.81	0.0030	0.724	Young vs. 10 months	-10.54	-21.81 to 0.72	0.0680
						Young vs. 22 months	-20.58	-31.84 to -9.30	0.0019
Fig. 2B	IL-6	Young, n=6; 10 months, n=4; 22 months, n=3	F(2,10)=17.94	0.0005	0.782	Young vs. 10 months	-9.937	-19.16 to -0.71	0.0371
						Young vs. 22 months	-23.45	-33.55 to-13.35	0.0003
Fig. 2C	TNFα	Young, n=6; 10 months, n=4; 22 months, n=3	F(2,10)=20.44	0.0003	0.803	Young vs. 10 months	-7.026	-12.70 to -1.35	0.0185
						Young vs. 22 months	-15.32	-21.54 to -9.09	0.0002
Fig. 2D	SOD1	Young, n=6; 10 months, n=4; 22 months, n=3	F(2,10)=7.220	0.0115	0.591	Young vs. 10 months	-1.318	-2.879 to 0.243	0.1004
						Young vs. 22 months	-2.462	-4.172 to -0.752	0.0078
Fig. 2E	SOD2	Young, n=6; 10 months, n=4; 22 months, n=3	F(2,10)=9.682	0.0046	0.659	Young vs. 10 months	-4.058	-8.100 to -0.016	0.0508
						Young vs. 22 months	-7.346	-11.77 to -2.92	0.0032
Fig. 2F	CAT	Young, n=5; 10 months, n=5; 22 months, n=4	F(2,11)=3.899	0.0525	0.415	Young vs. 10 months	2.097	-0.105 to 4.504	0.0883
						Young vs. 22 months	2.572	0.0185 to 5.125	0.0488
Fig. 2G	ROS Level	Young, n=3; 10 months, n=3; 22 months, n=3	F(2,6)=1.340	0.3303	0.309	Young vs. 10 months	-3.714	-12.13 to 4.700	0.3990
						Young vs. 22 months	-4.506	-12.92 to 3.909	0.2861

Note: Statistical parameters include one-way ANOVA *F* statistics, overall *P* values, eta squared effect sizes (η²), post hoc comparisons, mean differences, 95% confidence intervals (CIs), and adjusted *P* values for quantitative outcomes in the seminal vesicle. Data were analyzed using one-way ANOVA followed by Dunnett's multiple-comparisons test. Mean differences were calculated as the Young group minus the corresponding BBP-exposed group. Minor variations in sample size (*n*) across targets reflect the actual number of biological replicates included in each analysis based on sample availability and quality control. Abbreviations: CI, confidence interval; η², eta squared; ns, not significant ($P \geq 0.05$).

Supplementary Table S3. Detailed statistical parameters for quantitative outcomes in the testis.

Figure	Outcome	Groups, *n*	ANOVA	*P*-value	η²	Post hoc comparison	Mean difference	95% CI	Adjusted *P*
Fig. 3A	IL-1β	Young, n=6; Old, n=4; 10 months, n=5; 22 months, n=3	F(3,14)=5.563	0.0100	0.544	Old vs. Young	-3.745	-6.338 to -1.153	0.0052
						Old vs. 10 months	-1.202	-3.896 to 1.492	0.5178
						Old vs. 22 months	-1.101	- 4.169 to 1.966	0.6664
Fig. 3B	TNFα	Young, n=3; Old, n=4; 10 months, n=5; 22 months, n=4	F(3,12)=0.971	0.4383	0.195	Old vs. Young	-1.481	-11.51 to 8.544	0.9591
						Old vs. 10 months	-4.568	-13.37 to 4.237	0.4016
						Old vs. 22 months	0.3625	-8.919 to 9.644	0.9991
Fig. 3C	IL-6	Young, n=5; Old, n=3; 10 months, n=3; 22 months, n=4	F(3,11)=0.877	0.4823	0.193	Old vs. Young	4.525	-3.547 to 12.60	0.3315
						Old vs. 10 months	1.272	-7.754 to 10.30	0.9596
						Old vs. 22 months	2.836	-5.607 to 11.28	0.6847
Fig. 3D	SOD1	Young, n=4; Old, n=4; 10 months, n=5; 22 months, n=4	F(3,13)=0.534	0.6668	0.110	Old vs. Young	-0.3780	-1.338 to 0.5824	0.6034
						Old vs. 10 months	0.0185	-0.8927 to 0.9297	0.9999
						Old vs. 22 months	-0.0870	-1.047 to 0.8734	0.9897
Fig. 3E	SOD2	Young, n=3; Old, n=3; 10 months, n=5; 22 months, n=3	F(3,10)=1.540	0.2643	0.316	Old vs. Young	-1.083	-2.654 to 0.4878	0.1931
						Old vs. 10 months	-0.1449	-1.550 to 1.260	0.9825
						Old vs. 22 months	-0.5170	-2.088 to 1.054	0.6901
Fig. 3F	CAT	Young, n=6; Old, n=3; 10 months, n=5; 22 months, n=4	F(3,14)=8.496	0.0018	0.646	Old vs. Young	-2.363	-3.933 to -0.794	0.0036
						Old vs. 10 months	-0.381	-2.002 to 1.240	0.8496
						Old vs. 22 months	-0.273	-1.968 to 1.422	0.9410
Fig.3G	ROS Level	Young, n=6; Old, n=3; 10 months, n=3; 22 months, n=4	F(3,12)=0.783	0.5258	0.164	Old vs. Young	1.004	-8.531 to 10.54	0.9819
						Old vs. 10 months	-1.266	-12.28 to 9.743	0.9768
						Old vs. 22 months	4.223	-6.075 to 14.52	0.5538

Note: Statistical parameters include one-way ANOVA *F* statistics, overall *P* values, eta squared effect sizes (η²), post hoc comparisons, mean differences, 95% confidence intervals (CIs), and adjusted *P* values for quantitative outcomes in the testis. Data were analyzed using one-way ANOVA followed by Dunnett's multiple-comparisons test. Mean differences were calculated as the Old control group minus the corresponding comparison group. Minor variations in sample size (*n*) across targets reflect the actual number of biological replicates included in each analysis based on sample availability and quality control. Abbreviations: CI, confidence interval; η², eta squared; ns, not significant ($P \geq 0.05$).

Supplementary Table S4. Detailed statistical parameters for quantitative outcomes in TM3 Leydig cell experiments.

Figure	Outcome	Groups, *n*	ANOVA	*P*-value	η²	Post hoc comparison	Mean difference	95% CI	Adjusted *P*
Fig. 5B	FOXO1	CON, n=3; BBP 5 μM, n=4; BBP 50 μM, n=4	F(2,7)=4.385	0.0583	0.556	CON vs. 5 μM	-0.3923	-0.8556 to 0.0711	0.0901
						CON vs. 50 μM	0.0540	-0.4413 to 0.5493	0.9357
Fig. 5C	FOXO3	CON, n=4; BBP 5 μM, n=4; BBP 50 μM, n=4	F(2,9)=3.934	0.0592	0.466	CON vs. 5 μM	0.1658	-0.1594 to 0.4909	0.3488
						CON vs. 50 μM	0.3488	0.0236 to 0.6739	0.0369
Fig. 5D	FOXO6	CON, n=4; BBP 5 μM, n=3; BBP 50 μM, n=4	F(2,8)=4.029	0.0616	0.502	CON vs. 5 μM	-0.2620	-0.6915 to 0.1675	0.2384
						CON vs. 50 μM	-0.4188	-0.8164 to -0.0211	0.0408

Note: Statistical parameters include one-way ANOVA *F* statistics, overall *P* values, eta squared effect sizes (η²), post hoc comparisons, mean differences, 95% confidence intervals (CIs), and adjusted *P* values for protein expression levels in TM3 Leydig cells. Data were analyzed using one-way ANOVA followed by Dunnett's multiple-comparisons test. Mean differences were calculated as the control group (CON; 0.1% DMSO) minus the corresponding BBP-treated group (5 or 50 μM). Minor variations in sample size (*n*) across targets reflect the actual number of biological replicates included in each analysis based on sample availability and quality control. Abbreviations: CI, confidence interval; CON, control; η², eta squared; ns, not significant ($P \geq 0.05$).
